# The poplar Phi class glutathione transferase: expression, activity and structure of GSTF1

**DOI:** 10.3389/fpls.2014.00712

**Published:** 2014-12-23

**Authors:** Henri Pégeot, Cha San Koh, Benjamin Petre, Sandrine Mathiot, Sébastien Duplessis, Arnaud Hecker, Claude Didierjean, Nicolas Rouhier

**Affiliations:** ^1^Interactions Arbres - Microorganismes, Université de Lorraine, UMR1136Vandoeuvre-lès-Nancy, France; ^2^INRA, Interactions Arbres - Microorganismes, UMR1136Champenoux, France; ^3^Faculté des Sciences et Technologies, Université de Lorraine, CRM^2^, Equipe BioMod, UMR 7036Vandoeuvre-lès-Nancy, France; ^4^Faculté des Sciences et Technologies, CNRS, CRM^2^, Equipe BioMod, UMR 7036Vandoeuvre-lès-Nancy, France

**Keywords:** glutathione transferase, protein structure, crystallography, *Populus*, enzyme characterization, transcript profiling

## Abstract

Glutathione transferases (GSTs) constitute a superfamily of enzymes with essential roles in cellular detoxification and secondary metabolism in plants as in other organisms. Several plant GSTs, including those of the Phi class (GSTFs), require a conserved catalytic serine residue to perform glutathione (GSH)-conjugation reactions. Genomic analyses revealed that terrestrial plants have around ten GSTFs, eight in the *Populus trichocarpa* genome, but their physiological functions and substrates are mostly unknown. Transcript expression analyses showed a predominant expression of all genes both in reproductive (female flowers, fruits, floral buds) and vegetative organs (leaves, petioles). Here, we show that the recombinant poplar GSTF1 (PttGSTF1) possesses peroxidase activity toward cumene hydroperoxide and GSH-conjugation activity toward model substrates such as 2,4-dinitrochlorobenzene, benzyl and phenetyl isothiocyanate, 4-nitrophenyl butyrate and 4-hydroxy-2-nonenal but interestingly not on previously identified GSTF-class substrates. In accordance with analytical gel filtration data, crystal structure of PttGSTF1 showed a canonical dimeric organization with bound GSH or 2-(N-morpholino)ethanesulfonic acid molecules. The structure of these protein-substrate complexes allowed delineating the residues contributing to both the G and H sites that form the active site cavity. In sum, the presence of GSTF1 transcripts and proteins in most poplar organs especially those rich in secondary metabolites such as flowers and fruits, together with its GSH-conjugation activity and its documented stress-responsive expression suggest that its function is associated with the catalytic transformation of metabolites and/or peroxide removal rather than with ligandin properties as previously reported for other GSTFs.

## Introduction

Glutathione transferases (GSTs; EC 2.5.1.18) represent a ubiquitous multigenic family of enzymes that conjugate the reduced tripeptide glutathione (GSH, γ-Glu-Cys-Gly) on a wide range of endogenous and exogenous electrophilic molecules (Hayes et al., 2005). From the most recent genomic and phylogenetic analyses, the GST family is subdivided into 14 classes in photosynthetic organisms: Phi (F), Tau (U), Theta (T), Zeta (Z), Lambda (L), Hemerythrin (H), Iota (I), Ure2p, glutathionyl-hydroquinone reductase (GHR), elongation factor 1B Gamma (EF1Bγ), dehydroascorbate reductase (DHAR), tetrachlorohydroquinone dehalogenase (TCHQD), metaxin, microsomal prostaglandin E synthase type 2 (mpges-2) (Lallement et al., 2014a). Behind Tau GSTs, Phi GSTs (GSTFs) represent the second largest class in plants and this expansion probably results from several rounds of gene duplication (Lan et al., 2009). This class is often presented in the literature as plant-specific, however, basidiomycetes also possess GSTFs (Morel et al., 2013).

Along with GSTUs, plant GSTFs have been extensively studied for their involvement in herbicide detoxification and for this reason they could be considered as the counterparts of the mammalian drug metabolizing GSTs. By catalyzing GSH-conjugation reactions of electrophilic molecules that are subsequently recognized by vacuolar ABC transporters, GSTFs participate to the vacuolar sequestration and thus detoxification of exogenous compounds. However, other biochemical activities can account for the observed increased herbicide resistance. For instance, it was shown that the GSTF1 from the black grass *Alopecurus myosuroides*, a weed of cereals, possesses a glutathione peroxidase activity which lowers the levels of hydroperoxides produced in response to herbicides (Cummins et al., 1999). *Arabidopsis thaliana* transgenic plants expressing this GSTF1 acquire multiple herbicide resistance and accumulate protective flavonoids as initially observed in the black grass (Cummins et al., 2009, 2013). Another facet of GSTs is their involvement in secondary metabolism, in stress response and in their associated signaling. For instance, *A. thaliana* GSTF6 is required for the synthesis of the defense compound camalexin, by catalyzing the conjugation of glutathione onto indole-3-acetonitrile (Su et al., 2011) whereas *A. thaliana* GSTF2 binds tightly to camalexin and might be required for its transport (Dixon et al., 2011). On the other hand, *A. thaliana* GSTF8 catalyzes glutathione conjugation to prostaglandin 12-oxophytodienoic acids and A_1_-phytoprostanes, two stress signaling molecules (Mueller et al., 2008). Consistent with these functions, the expression of *GST* gene belonging to all classes is often highly induced in response to biotic and abiotic stresses or to hormone treatments, and this often correlated with an increase in the protein amount. For instance, the expression of several *GSTF* genes is enhanced in response to plant hormones such as ethylene, methyl jasmonate, salicylic acid and auxin, to herbicides and to herbicide safeners, to pathogen infection, and more generally to treatments leading to oxidative stress (Deridder et al., 2002; Wagner et al., 2002; Lieberherr et al., 2003; Smith et al., 2003, 2004; Sappl et al., 2004, 2009).

Interestingly, previous biochemical analyses have shown that GSTFs can bind to metabolites for non-catalytic functions. The best characterized example of carrier/transport functions for a Phi GST concerns the requirement of *A. thaliana* transparent testa 19 (tt19)/AtGSTF12 and of the petunia ortholog AN9 for the correct vacuolar localization of anthocyanins and pro-anthocyanidins (Alfenito et al., 1998; Kitamura et al., 2004). While it was initially thought that these GSTFs could catalyze GSH-conjugation reactions, it was determined that they serve as flavonoid carrier proteins (Mueller et al., 2000). Moreover, photoaffinity-labeling experiments or competition activity assays pointed to the capacity of GSTFs to bind plant hormones such as gibberellic acid (Axarli et al., 2004), cytokinin and auxin (Bilang et al., 1993; Bilang and Sturm, 1995; Gonneau et al., 2001). A screen for metabolites able to bind to *A. thaliana* GSTF2, either from pure molecules or from plant or *Escherichia coli* extracts also identified other interacting molecules. Besides camalexin, flavonoids (quercetin, quercetin-3-O-rhamnoside and kaempferol) and other heterocyclic compounds structurally close to flavonoids (harmane, norharmane, indole-3-aldehyde, and lumichrome) have been shown to bind to AtGSTF2 (Smith et al., 2003; Dixon et al., 2011). The absence of GSH-conjugation activity with these compounds indicated that AtGSTF2 functions as a carrier protein. Moreover, competition binding experiments or activity assays in the presence of several of these binding molecules showed that they either did not alter AtGSTF2 conjugating activity or even increased it, hinting the existence of multiple ligand/substrate binding sites.

At the structural level, GSTFs exist as homodimers, the dimerization interface involving mainly hydrophobic surface patches (Armstrong, 1997). Each monomer comprises an active site region formed by a glutathione binding pocket (G-site) primarily involving residues from the conserved N-terminal thioredoxin domain and an hydrophobic pocket (H-site) primarily involving residues from the less conserved C-terminal domain (Prade et al., 1998). In their active sites, most GSTFs present a serine residue that is located in the N-terminal end of the α1 helix which promotes the formation of the active thiolate anion on the sulphydryl group of the cysteine of GSH that is required for catalysis. However, the non-catalytic functions observed for some GSTFs suggested the existence of a ligandin site (L-site) but structural details of the latter are still lacking. From mutagenesis experiments performed on *Zea mays* GST-I, the L-site is likely overlapping with the G- and H-sites (Axarli et al., 2004).

In this study, the transcript levels of the eight poplar *GSTFs* have been analyzed in various organs. Then, the biochemical and structural properties of the stress-responsive GSTF1 have been further characterized, examining the enzymatic properties of recombinant proteins (WT protein and variants mutated for the catalytic serine) and solving the 3D structure of the protein in complex with substrates/ligands.

## Materials and methods

### Genomic and phylogenetic analyses

In order to identify all poplar *GSTF* genes, homology searches with the BLAST algorithm have been performed on the different versions of the *P. trichocarpa* genome including the version 3.0 available on the phytozome v10 portal (http://phytozome.jgi.doe.gov/pz/portal.html). Genome analyses for other terrestrial plants have been also performed on the phytozome v10 portal whereas cyanobacterial and algal genomes have been analyzed from cyanobase (http://genome.microbedb.jp/cyanobase) and the JGI genome portal (http://genome.jgi.doe.gov) respectively. The protein sequences and corresponding accession numbers can be found as Supplementary Table 1. When possible, GSTF sequences were corrected on the basis of available ESTs.

### Biological material, growth conditions, and inoculation procedures

Hybrid poplar cultivar ‘Beaupré’ (*Populus trichocarpa* × *Populus deltoides*) greenhouse cultivation, *Melampsora larici-populina* urediniospore multiplication and leaf inoculation procedures were done as previously described (Rinaldi et al., 2007). The *M. larici-populina* isolates used in this study are 98AG31 (pathotype 3-4-7) and 93ID6 (pathotype 3-4) respectively virulent and avirulent on “Beaupré.” Poplar organs have been harvested from a naturally growing male and female *P. trichocarpa* adult trees found on the faculty of sciences campus located in Vandoeuvre-lès-Nancy (France).

### RT-PCR experiments

Total RNAs were extracted from 150 mg of *P. trichocarpa* stamens, male flowers, female flowers, fruits, petioles, leaves, buds, and roots using the RNeasy Plant Mini Kit (Qiagen) according to the Manufacturer's instructions with minor modifications described before (Lallement et al., 2014b). Then, mRNAs were reverse-transcribed to obtain cDNAs by using the iScript cDNA Synthesis kit (Bio-Rad) following the manufacturer's instructions. PCR amplifications were performed for 25, 30, or 35 cycles using Go-Taq polymerase (Promega). Specific forward and reverse primers (Supplementary Table 2) have been designed to amplify *ca* 300 bp fragments of each *GSTF* gene. The ubiquitin gene (Potri.015G013600) was used as a control of the cDNA concentration used for PCR amplification and incidentally of cDNA integrity (Lallement et al., 2014b). The PCR products have been separated by electrophoresis on 1% agarose gel and visualized by ethidium bromide staining.

### Protein extraction and western-blot analysis

Extraction of soluble proteins from leaves, petioles, stems, roots, fruits, stamens, and buds or from rust-infected leaves was performed as previously described (Vieira Dos Santos et al., 2005). The proteins were separated by 15% SDS–PAGE and electro-transferred onto nitrocellulose membranes (LI-COR Biosciences). After rinsing in 13.7 mM NaCl, 0.27 mM KCl, 10 mM Na_2_HPO_4_, and 0.2 mM KH_2_PO_4_ buffer (phosphate buffered saline: PBS), membranes were blocked during 45 min at room temperature using the Odyssey blocking buffer (LI-COR Biosciences). Then, membranes were incubated with rabbit polyclonal antibodies (diluted 1:1000, synthesis by Genecust) raised against PttGSTF1 for 30 min in the presence of 0.05% of tween 20. After several washing steps with a PBS buffer supplemented with 0.05% tween 20 (PBST), membranes were incubated for 30 min with IRDye 800 CW goat or donkey anti-rabbit secondary antibodies (LI-COR Biosciences) diluted 1:5000 in the Odyssey blocking buffer supplemented with 0.05% tween 20 and 0.01% SDS. After extensive washes with PBST and PBS, immunodetection of proteins on the membrane was performed by exciting the IRDye with an Odyssey Infrared Imager (LI-COR Biosciences).

### PCR cloning and site-directed mutagenesis

The sequence coding for GSTF1 was amplified by PCR from *Populus tremula* × *P. tremuloides* leaf cDNAs using specific forward and reverse primers (Supplementary Table 2) and cloned into pET-3d between *Nco*I and *Bam*HI restriction sites. Hence, the sequence is subsequently referred to as PttGSTF1. PttGSTF1 S13C and PttGSTF1 S13A variants where the serine found at position 13 is substituted into cysteine or alanine were generated by site-directed mutagenesis using two complementary mutagenic primers (Supplementary Table 2). Two overlapping mutated fragments were generated in a first PCR reaction and were subsequently used in a second PCR to generate the full-length mutated sequences which have been then cloned into pET-3d.

### Heterologous expression in *E. coli* and purification

PttGSTF1 expression was performed in an *E. coli* BL21 (DE3) strain (Novagen) containing the pSBET plasmid upon transformation with the recombinant pET-3d plasmids. Bacteria were cultivated at 37°C in LB medium containing kanamycin (50 μg/ml) and ampicillin (50 μg/ml). When the cell culture reached an OD_600nm_ of 0.7, PttGSTF1 expression was induced by the addition of 0.1 mM isopropyl β-D-1-thiogalactopyranoside (IPTG) and cells were further grown for 4 h. Cells were harvested by centrifugation, resuspended in a 30 mM Tris-HCl pH 8.0, 1 mM EDTA, 200 mM NaCl buffer and stored at −80°C. Cell lysis was achieved by two rounds of 1 min sonication. The cell extract was then centrifuged at 40,000 g for 30 min at 4°C to remove cellular debris and aggregated proteins. The fraction precipitating between 40 and 80% of the saturation in ammonium sulfate was subjected to a size-exclusion chromatography by loading the protein extract on an Ultrogel^®^ ACA44 (5 × 75 cm, Biosepra) column equilibrated with 30 mM Tris-HCl pH 8.0, 200 mM NaCl buffer. The fractions containing the recombinant protein were then pooled, dialyzed by ultrafiltration in Amicon cells using a YM10 membrane (Millipore) and loaded onto a DEAE-cellulose column (Sigma Aldrich) equilibrated in 30 mM Tris-HCl pH 8.0. The proteins were eluted using a 0–400 mM NaCl gradient, concentrated by ultrafiltration and stored in 30 mM Tris-HCl pH 8.0, 200 mM NaCl buffer. The protein purity was then analyzed by 15% SDS-PAGE and protein concentration was determined after measuring the absorbance at 280 nm using a theoretical molar absorption coefficient of 33,982 M^−1^ cm^−1^ for PttGSTF1, PttGSTF1 S13C, and PttGSTF1 S13A.

### Determination of the molecular mass and oligomerization state of purified recombinant proteins

The molecular masses of purified recombinant proteins were analyzed using a Bruker microTOF-Q spectrometer (Bruker Daltonics, Bremen, Germany) equipped with an Apollo II electrospray ionization source as described previously (Couturier et al., 2011). The oligomerization state of purified recombinant proteins was analyzed on a Superdex 200 10/300 column equilibrated in 30 mM Tris-HCl pH 8.0, 200 mM NaCl and connected to an Akta purifier system (GE Healthcare) by injecting 100 μg of purified recombinant proteins at a flow rate of 0.5 ml/min. The column was calibrated using the molecular weight standards (6500–700,000 Da) from Sigma.

### Enzymatic activities

The GSH-conjugation activity toward phenetyl isothiocyanate (PITC), benzyl isothiocyanate (BITC), 1-chloro-2,4-dinitrobenzene (CDNB), 4-hydroxy-2-nonenal (HNE), 4-nitrophenyl butyrate (PNP-butyrate) was assayed at 25°C by following absorbance at 274 nm for isothiocyanate derivatives, or at 224, 340, 412 nm for HNE, CDNB, and PNP-butyrate respectively. Reactions were carried out in 500 μL of 100 mM phosphate buffer pH 6.5 for both isothiocyanate derivatives and HNE; 100 mM sodium phosphate buffer pH 7.5 for PNP-butyrate and 30 mM Tris-HCl pH 8.0, 1 mM EDTA for CDNB. Various concentrations of PITC (50–500 μM), HNE (12.5–125 μM), CDNB (500–6000 μM), BITC (100–1000 μM) or PNP-butyrate (50–3000 μM) have been tested at a fixed GSH concentration of 1 mM. When using HNE as a substrate, the GSH concentration was fixed at 0.7 mM to limit interferences with the detection of HNE at 224 nm.

Thiol-transferase, dehydroascorbate (DHA) reductase and peroxidase activities have been measured toward 2-hydroxyethyl disulfide (HED), DHA, and cumene hydroperoxide (CuOOH) or tert-butyl hydroperoxide (t-BOOH) respectively using an NADPH-coupled spectrophotometric method. The reactions were carried out at 25°C in 500 μL of 30 mM Tris-HCl, pH 8.0, 1 mM EDTA buffer containing 150 μM NADPH, 0.5 units of yeast glutathione reductase and various concentrations of HED (25–1000 μM), DHA (250–5000 μM), CuOOH (500–6000 μM), t-BOOH (250–5000 μM) at a fixed GSH concentration of 2 mM.

For all these assays, reactions were started by the addition of the enzyme and protein concentrations used were within the linear response range. The measured velocities were corrected by subtracting the rate of spontaneous non-enzymatic reaction and three independent experiments were performed at each substrate concentration. Changes in absorbance were followed with a Cary 50 spectrophotometer (Agilent Technologies). The kinetic parameters (k_cat_ and apparent K_m_) were obtained by fitting the data to the non-linear regression Michaelis–Menten model in GraphPad Prism 5 software. The k_cat_ values are expressed as μmol of substrate oxidized per second per μmol of enzyme (i.e., the turnover number in s^−1^), using specific molar absorption coefficients of 6220 M^−1^ cm^−1^ at 340 nm for NADPH, 8890 M^−1^ cm^−1^ at 274 nm for PITC, 9250 M^−1^ cm^−1^ at 274 nm for BITC, 9600 M^−1^ cm^−1^ at 340 nm for CDNB, 17700 M^−1^ cm^−1^ at 412 nm for PNP-butyrate and 13750 M^−1^ cm^−1^ at 224 nm for HNE.

### Crystallization and structure determination of PttGSTF1 and PttGSTF1 S13C

Initial screening of crystallization conditions was carried out by the microbatch-under-oil method. Sitting drops were set up using 1 μl of a 1:1 mixture of protein and crystallization solutions (672 different commercially available conditions) in Terasaki microbatch multiwell plates (Molecular Dimensions). The crystallization plates were stored at 4°C. Single crystals of sufficient size were obtained using Jena Bioscience 2D1 condition (30% w/v PEG 4000, 100 mM 2-(N-morpholino)ethanesulfonic acid (MES) sodium salt, pH 6.5). Best crystals were obtained with a protein concentration of 14 mg/ml for PttGSTF1 and of 10 mg/ml for PttGSTF1 S13C. The single crystals were flash-cooled in liquid nitrogen using a mixture of the crystallization solution and 20% glycerol as cryoprotectant. For PttGSTF1, before crystallization, the protein (*ca* 1 mL at 600 μM) was treated with 10 mM GSH for 30 min, desalted on G25 columns and concentrated to the indicated concentration using Amicon Ultra centrifugal filters, Ultracel 10 K Membrane from Millipore.

PttGSTF1 X-ray diffraction data were collected on beamline EMBL-X11 at the DORIS storage ring (DESY, Hamburg, Germany) and PttGSTF1 S13C X-ray diffraction data were collected on beamline BM30A at synchrotron ESRF (Grenoble, France). PttGSTF1 and PttGSTF1 S13C diffraction images were integrated with the program HKL2000 (Otwinowski and Minor, 1997) and the program XDS (Kabsch, 2010), respectively. Crystallographic calculations were carried out with programs from the CCP4 program suite (Winn et al., 2011). The structure of PttGSTF1 was solved by the molecular replacement method with the program Molrep (Vagin and Teplyakov, 2010) using *A. thaliana* GSTF2 as a template (PDB code: 1GNW). PttGSTF1 and PttGSTF1 S13C structures were refined by alternate cycles of restrained maximum-likelihood refinement with the program Phenix (Adams et al., 2010) and manual adjustments were made to the models with Coot (Emsley et al., 2010). The crystal parameters, data statistics, and final refinement parameters are shown in Table 1. All structural figures were generated with PyMol Molecular Graphics System (Schrödinger, LLC). The atomic coordinates and structure factors (codes 4RI6 and 4RI7 for PttGSTF1 and PttGSTF1 S13C, respectively) have been deposited in the Protein Data Bank, Research Collaboratory for Structural Bioinformatics, Rutgers University, New Brunswick, NJ (http://www.rcsb.org/).

**Table 1:** **Statistics of X-ray diffraction data collection and model refinement**.

	**PttGSTF1**	**PttGSTF1 S13C**
**DATA COLLECTION**		
Space group	*P*2_1_2_1_2_1_	
Nb of monomers in the ASU^a^	2	
Cell dimensions a, b, c (Å)	58.84 65.94 109.84	55.30 60.65 119.90
Resolution (Å)	18.28-1.52 (1.55-1.52)^b^	40.86-1.80 (1.90-1.80)
Rmerge	0.034 (0.20)	0.124 (0.614)
Mean I/σ (I)	40.6 (8.4)	11.7 (2.1)
Completeness (%)	99.2 (95.2)	96.5 (79.0)
*n* observations	7,26,145 (29,691)	2,41,669 (17,902)
Average redundancy	11.0 (9.0)	6.6 (4.2)
Wilson B factor (Å^2^)	19.4	14.8
**REFINEMENT**		
Resolution (Å)	18.28-1.52 (1.54-1.52)	40.86-1.80 (1.85-1.80)
*n* reflections	65,579 (2554)	36,817 (2118)
Cutoff	*F* > 0σ(*F*)	*F* > 0σ(*F*)
Rall (%)^c^	15.3	15.3
Rfree (%)^c^	18.3 (19.6)	19.5 (28.9)
Average B-factor (Å^2^)		
Protein atoms	24.2	18.5
Ligand atoms	25.8	17.2
Solvent atoms	35.8	29.2
Ramachandran statistics (%)		
Residues in preferred regions	98.6	97.9
Residues in allowed regions	1.2	1.9
Outlier residues	0.2	0.2
R.m.s.^d^ deviations		
Bond length (Å)	0.009	0.01
Bond angle (°)	1.26	1.3

a ASU, Asymmetric unit.

b Values in parentheses are for highest resolution shell.

c Rall was determined from all the reflections (working set + test set) whereas Rfree corresponds to a subset of reflections (test set).

d R.m.s.: Root mean square.

## Results

### Phylogenetic and sequence analyses of *P. trichocarpa* GSTFs

**Figure 1:**
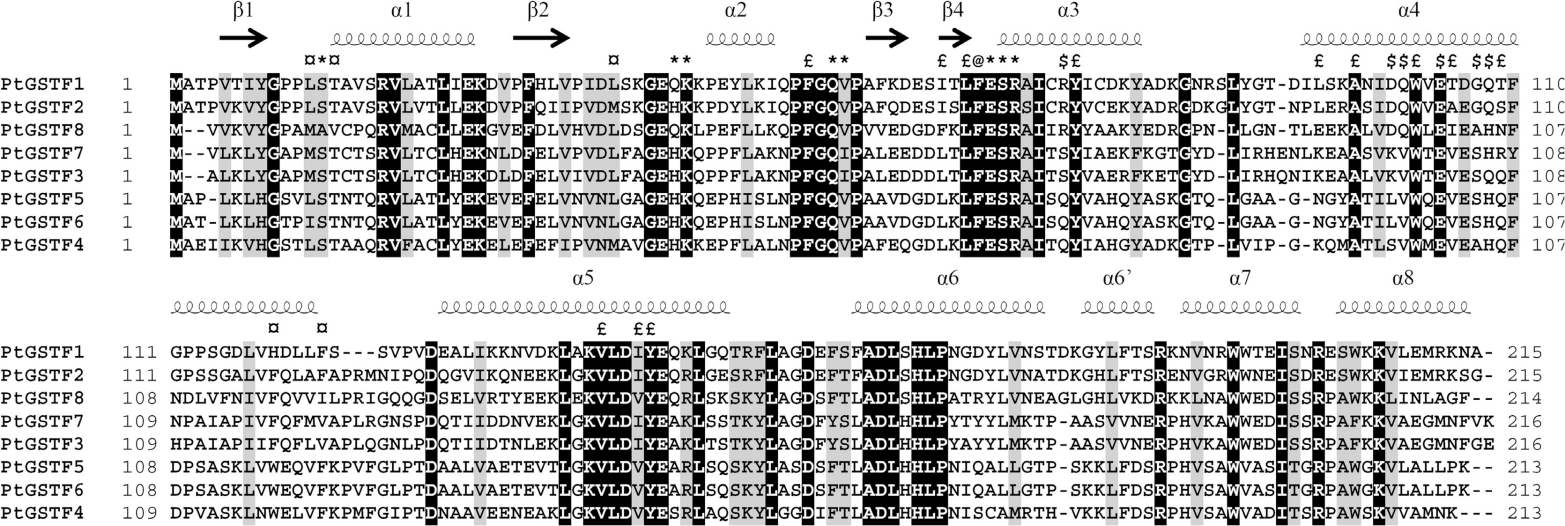
**Structure based sequence alignment of *Populus trichocarpa* GSTFs**. The structural alignment was calculated with PROMALS3D using the structure of PttGSTF1 and rendered using ESpript 3.0. Accession numbers of GSTFs from the version 3.0 of *P. trichocarpa* genome are the following: PtGSTF1: Potri.002G015100, PtGSTF2: Potri.002G015200, PtGSTF3: Potri.014G132200, PtGSTF4: Potri.T035400, PtGSTF5: Potri.T035300, PtGSTF6: Potri.T035100, PtGSTF7: Potri.T035000, PtGSTF8: Potri.017G138800. From the structure of PttGSTF1, residues contributing to the dimerization and those involved in the G- or H-sites are highlighted as follows: ^*^, glutathione-interacting residues; ¤, MES-interacting residues; $, dimer interface via hydrogen bond; £, dimer interface via van der Waals interactions; @, dimer interface via hydrogen bond and van der Waals interactions.

**Figure 2:**
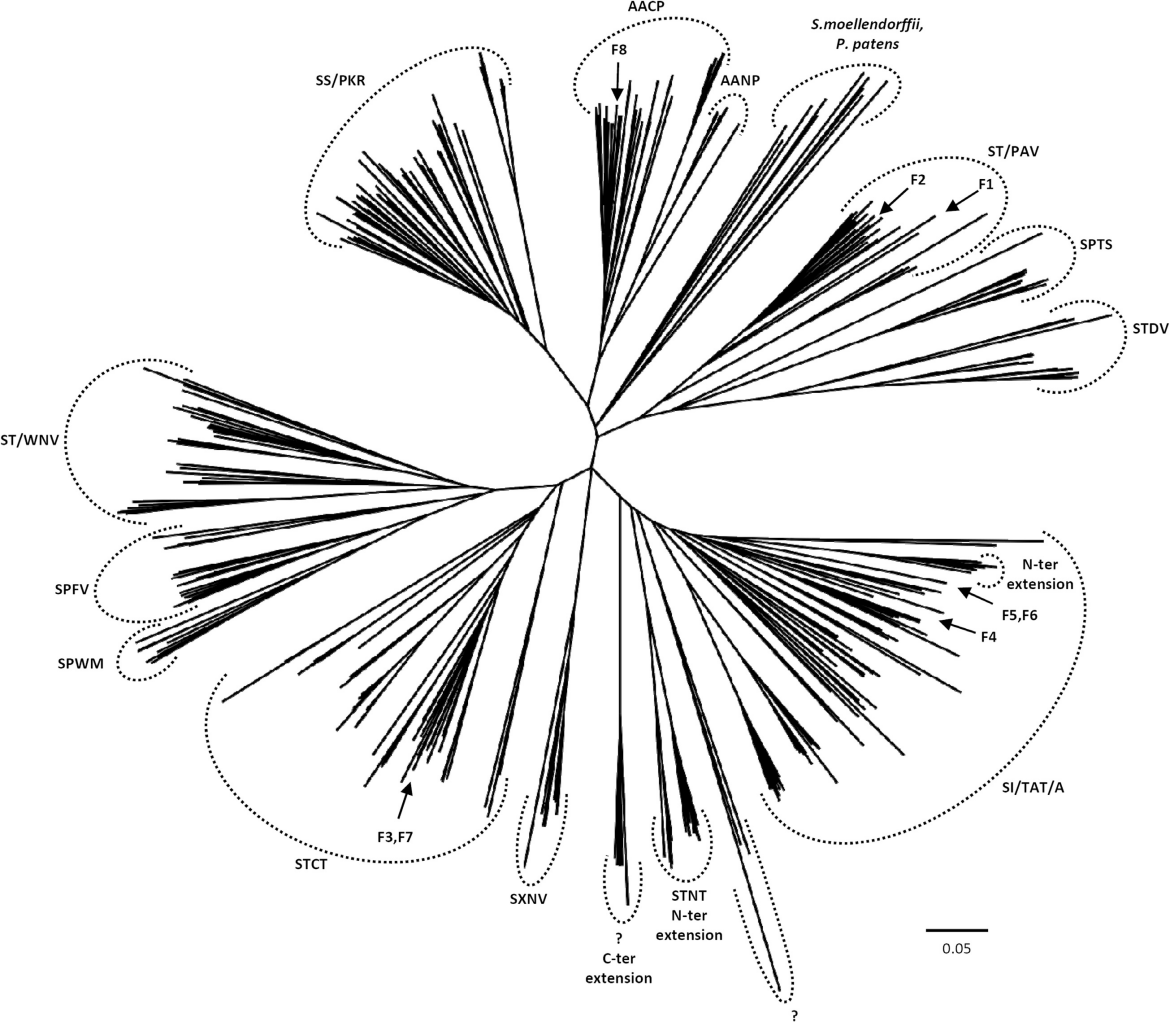
**Unrooted phylogenetic tree of GSTFs from terrestrial plants**. The alignment was performed with PROMALS3D using 1BYE, 1AXD, 1AW9, 1GNW, and 1BX9 protein structure models as templates. The alignment was subsequently manually adjusted by using Seaview software. Phylogenetic tree was built with BioNJ and edited with Figtree software (http://tree.bio.ed.ac.uk/software/figtree/). Five hundred bootstrap replicates were performed in order to test the robustness of the tree. The scale marker represents 0.05 substitutions per residue. Sequence names have been removed for clarity but all sequences used are available as Supplementary Table S1. For each major branch, the consensus active site signature containing the catalytic residue is indicated, x is used when the variability is too high. *P. trichocarpa* isoforms have been indicated by an arrow on the tree (F1–F8).

*In silico* analysis of the various versions of *P. trichocarpa* genome led to the identification of eight genes coding for GSTFs. All the *P. trichocarpa GSTF* genes encode predicted proteins with a size ranging from 213 to 218 amino acids (Figure 1). None of these sequences exhibits a targeting sequence, suggesting a cytosolic localization. Based on sequence similarities and phylogenetic analysis, four subgroups can be distinguished in poplar: PtGSTF1/2, PtGSTF3/7, PtGSTF4/5/6, and PtGSTF8 (Figures 1, 2). In terms of sequence similarity, the percentage identity within a subgroup ranges from 65 to 98% whereas it is comprised between 40 and 48% between subgroups. The protein similarity somehow reflects the gene arrangement since *PtGSTF1* and *PtGSTF2* genes are present in tandem on chromosome 2, *PtGSTF4*, 5, 6, and 7 genes cluster on the scaffold 36, whereas *PtGSTF3* and *PtGSTF8* are found at isolated loci on the chromosomes 14 and 17, respectively. Hence, the only peculiarity is the genomic association of *PtGSTF7* with *PtGSTF4*, *5*, *6* whereas the sequence proximity to *PtGSTF3* suggested a common origin. The sequence differences between members of each group are also visible by looking to the four amino acid signature typical of proteins of the thioredoxin superfamily and containing the catalytic serine. Indeed, PtGSTF1 and PtGSTF2 display a STAV active site motif, PtGSTF3 and 7 a STCT motif and PtGSTF4, 5, and 6 display STAA or STNT motifs (Figure 1). PtGSTF8 is clearly particular since it has an alanine (AVCP motif) instead of the catalytic serine. This is not specific to the poplar isoform as this particularity is found in several plant orthologs, including the petunia AN9 protein for example.

An exhaustive search of GSTF homologs in available genomes from photosynthetic organisms indicated that *GSTF* genes are absent in cyanobacteria and green algae. On the other hand, there are considerable variations in the number of genes in terrestrial plants since there is only one gene in *Selaginella moellendorffii* but 27 predicted genes in *Aquilegia coerulea* (Supplementary Table 1). However, the average number of genes is close to 10. A phylogenetic tree constructed using the 400 retrieved sequences (Figure 2) shows several distinct clades that can be distinguished according to the protein active site signature even though some groups can be also differentiated on the basis of the presence of C-terminal or N-terminal extensions. The sequences identified in *P. patens* and *S. moellendorffii*, which are supposed to represent the ancestral versions, form an isolated clade and do not display a clear consensus active site motif. The four subgroups observed for poplar GSTFs are found again in the phylogenetic tree and fell within separate clades. It is worth noting that PtGSTF8 stands out within a clade containing proteins lacking the catalytic serine but displaying a conserved cysteine residue two residues away (AxC motif). Interestingly, this cysteine is also found in PtGSTF3 and PtGSTF7 and in all orthologs of the same clade whereas the catalytic serine is present.

Overall this indicates that numerous species-specific duplication events occurred during evolution and this raises the question of the appearance of the GSTF group in photosynthetic organisms since it appears to be an innovation specifically found in terrestrial plants. Moreover, the divergences observed in the active site signatures suggest that the proteins may have different properties.

### Transcript expression of GSTFs in poplar organs

**Figure 3:**
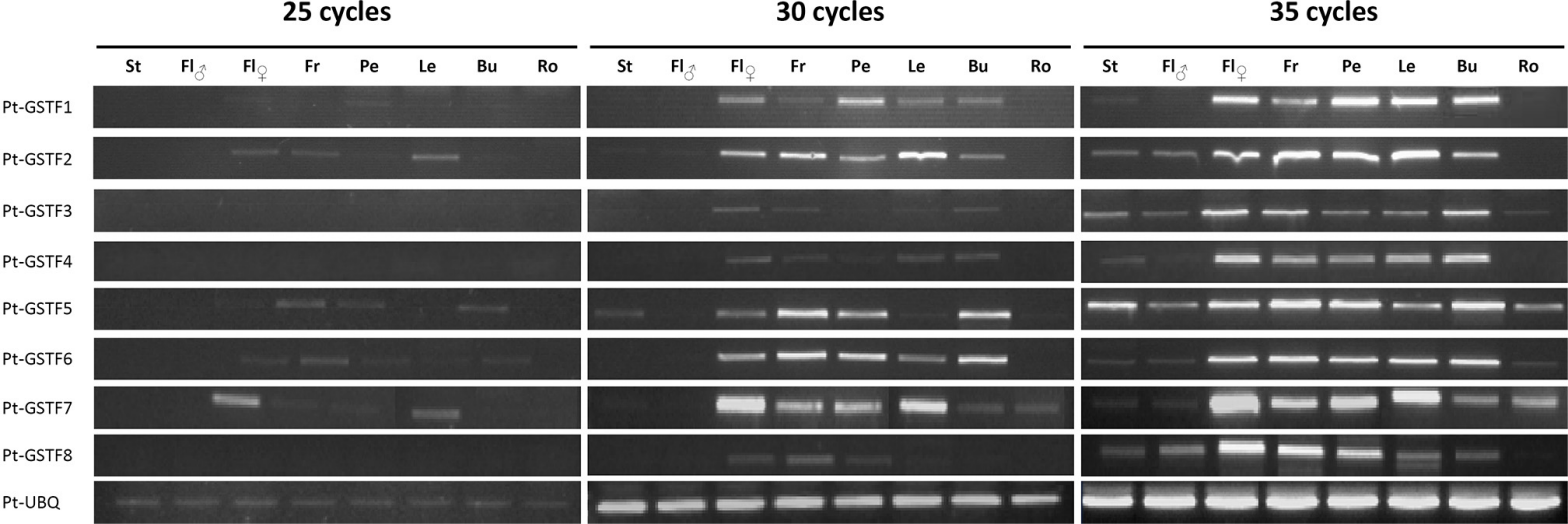
**Transcript accumulation of *GSTFs* in poplar organs**. RT-PCR experiments were performed using cDNAs from stamens (St), male flowers (Fl♂), female flowers (Fl♀), fruits (Fr), petioles (Pe), leaves (Le), buds (Bu), and roots (Ro). Ubiquitin was used as a reference gene.

In order to determine whether the expression territories could allow discriminating *GSTF* genes, RT-PCR experiments were performed from different tissues of an adult, naturally-growing *P. trichocarpa* individual. Experiments were performed with 25, 30, or 35 amplification cycles in order to examine gene expression in the linear range of PCR amplification. The best detection was obtained after 30 cycles as transcripts were barely detected at 25 cycles whereas the signal for some genes was saturated at 35 cycles. All GSTF transcripts were weakly detected in roots and in the male reproductive organ either as whole (male flower) or in stamen, whereas they were all detected in female flowers, fruits, petioles, leaves, and buds (Figure 3). Moreover, the *GSTF1*, *F2*, *F5*, *F6*, and *F7* genes are globally more expressed than *GSTF3*, *F4*, and *F8* genes. Comparing the expression of duplicated genes, we observed that they generally have the same expression profiles although variations in transcript abundance can sometimes be observed. A difference between *PtGSTF1* and *PtGSTF2* transcripts is the presence of *PtGSTF2* in male flowers. In the *PtGSTF4*/*F5/F6* subgroup and incidentally among all GSTFs tested, *PtGSTF5* is the most expressed in male flowers/stamen and in roots together with *PtGSTF7*.

### PtGSTF1 protein accumulates in all organs analyzed but its level is not affected in leaves infected by the rust fungal pathogen *Melampsora larici-populina*

In the subsequent parts, we focused our analysis on poplar GSTF1 since several studies showed that it is regulated in many stress conditions. For instance, it is up-regulated in poplar leaves exposed to the tent caterpillar *Malacosoma disstria* (Ralph et al., 2006), in root apices of drought-sensitive (Soligo) and tolerant (Carpaccio) poplar cultivars and in leaves of Carpaccio cultivar subjected to a water deficit (Cohen et al., 2010) and in leaves of 2 month-old *P. trichocarpa* cuttings treated with CDNB or H_2_O_2_ (Lan et al., 2009). Contrasting results have been obtained in the case of poplar infection by rust fungi, GSTF1 was found to be up-regulated in some (Miranda et al., 2007) but not all studies (Rinaldi et al., 2007; Azaiez et al., 2009). Besides, proteomic studies pointed to an increased GSTF1 protein level in roots of *Populus tremula* exposed to a cadmium stress (Kieffer et al., 2009) and in leaves of *Populus cathayana* male cuttings exposed to chilling or salt stresses (Chen et al., 2011; Zhang et al., 2012).

**Figure 4:**
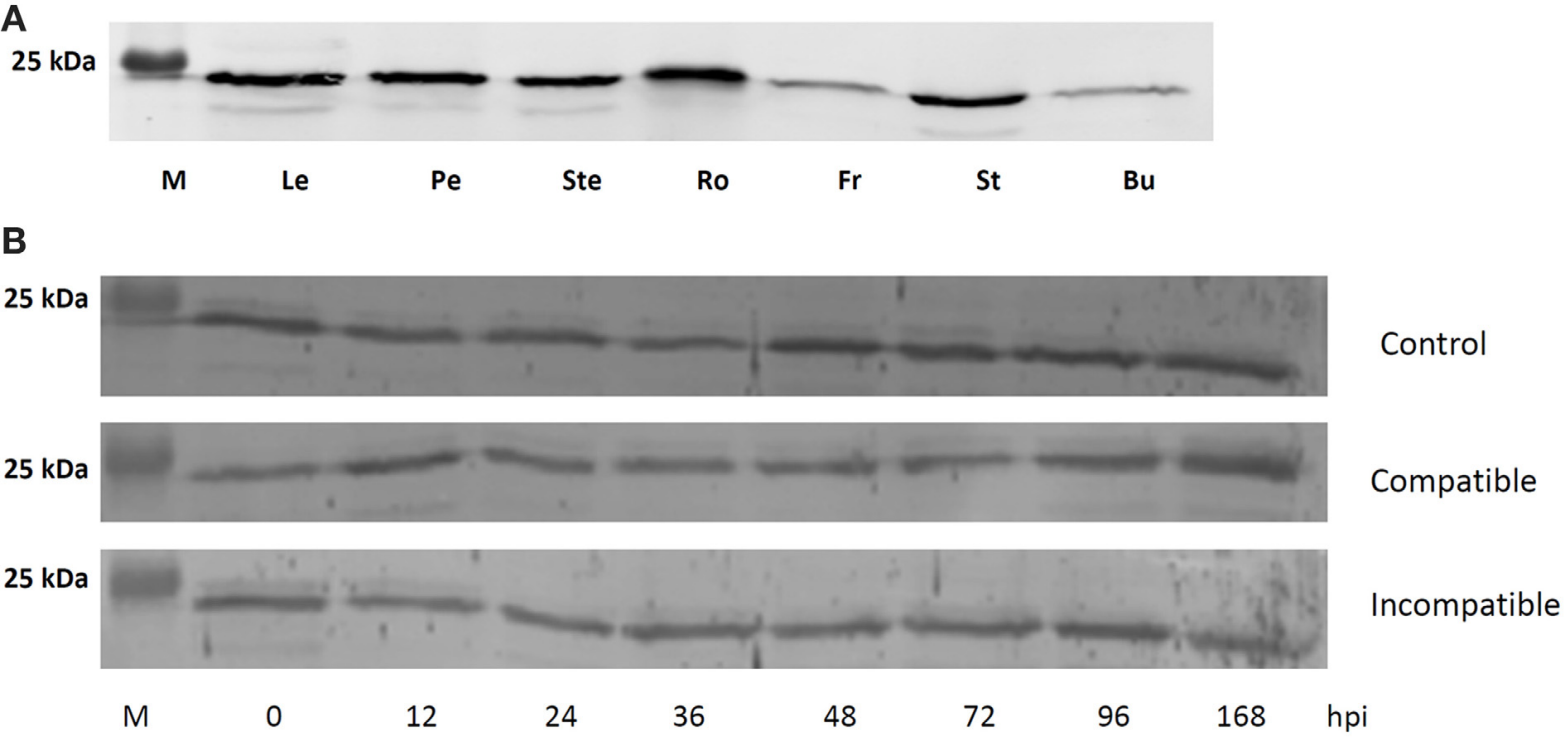
**GSTF1 protein abundance in poplar organs and in rust-infected leaves. (A)** Western blot analysis was performed from 30 μg of soluble protein extracts from leaves (Le), petioles (Pe), stems (Ste), roots (Ro), fruits (Fr), stamens (St), and buds (Bu). **(B)** Western blot analysis from 30 μg of soluble protein extracts from leaves infected or not with virulent or avirulent isolates of *M. larici populina* leading respectively to compatible or incompatible interactions. Control refers to as mock inoculated treatment. Time-points correspond to key developmental stages, i.e., penetration through the stomata (12 hpi), formation of the first haustorial infection structures (24 hpi), arrest of avirulent isolate growth (48 hpi) and formation of uredinia symptoms by the virulent isolate and urediniospores release (168 hpi).

Hence, taking advantage of the production of the recombinant protein (see below), we have raised an antibody against GSTF1 first to investigate its protein level in several poplar organs, i.e., leaves, petioles, stems, roots, fruits, stamens, and buds by Western Blotting (Figure 4A). A major band around 25 kDa likely corresponding to GSTF1 was detected in protein extracts from various organs, indicating that the protein is present in many tissues, though a higher protein amount was found in leaves, petioles, stems, roots and stamens. Considering that GSTF2 is a close paralog, it is possible that the detected signal represents the sum of both GSTFs. Next, considering the discrepancy observed at the transcript level as detailed above, we sought to evaluate GSTF1 protein abundance in a poplar-rust pathosystem. The model used is *P. trichocarpa* × *P. deltoides* leaves either untreated or inoculated by two *M. larici-populina* isolates, virulent, or avirulent, leading to compatible and incompatible reactions respectively (Figure 4B). However, no significant variation in protein abundance was detected over a 7-day time-course infection which represents a whole asexual cycle from spore germination to urediniospore formation. This result suggests that GSTF1 protein levels are not affected by *M. larici-populina* infections.

### Poplar GSTF1 is a homodimeric protein with GSH-conjugating activities

In order to investigate the biochemical and structural properties of GSTF1, the mature form was expressed in *E. coli* as well as single mutated protein variants, the catalytic serine of which was replaced by a cysteine or an alanine residue. Having used a *P. tremula* × *P. tremuloides* leaf cDNA library, the amplified coding sequence, which is perfectly similar to the DN500362 EST sequence, is slightly different from the GSTF1 version found in the *P. trichocarpa* reference genome. Hence, the sequence will be referred to as PttGSTF1 in the following parts for *P. tremula* × *P. tremuloides* GSTF1. At the protein level, two very conservative changes are present, Ile33 is replaced by a Val and Lys86 by an Arg. After purification, around 30 mg of protein was obtained per liter of culture.

The purified proteins have been first analyzed by mass spectrometry. A single species was detected for each protein with molecular masses of 24192, 24511, and 24172 Da for PttGSTF1, PttGSTF1 S13C, and PttGSTF1 S13A respectively (Supplementary Table 3). Compared to theoretical masses, these values are compatible with proteins where the N-terminal methionine is cleaved, which was expected form the presence of an alanine as the second residue. A mass increment of 305 Da was specifically present in PttGSTF1 S13C, which suggested that a glutathione molecule is covalently bound to the newly introduced cysteine residue via a disulfide bridge. Accordingly, PttGSTF1 S13C is not retained on GSH Sepharose columns contrary to PttGSTF1 and PttGSTF1 S13A. Then, the oligomeric state of wild-type and mutated proteins was estimated using calibrated size exclusion chromatography. All purified proteins eluted as a single peak whose estimated mass (45–47 kDa) is consistent with a dimeric arrangement (Supplementary Table 3) as reported for example for Arabidopsis GSTF2 or maize GST-I proteins (Reinemer et al., 1996; Neuefeind et al., 1997a).

Next, in order to characterize the enzymatic properties of PttGSTF1, its activity was measured toward various model substrates (CDNB, BITC, PITC, PNP-butyrate, and HNE) usually employed to measure the activities of GSTs catalyzing GSH-conjugation reactions (Table 2). An activity was detected toward all these substrates with catalytic efficiencies (k_cat_/K_m_) ranging from 6.6 × 10^2^ M^−1^ s^−1^ for PNP-butyrate to 3.1 × 10^3^ M^−1^ s^−1^ for HNE. The slightly better catalytic efficiency obtained for HNE compared to other substrates is due to a better affinity of PttGSTF1 for this substrate. On the other hand, the lower efficiency observed with PNP-butyrate is due to a weak turnover number (k_cat_). The kinetic parameters for the two tested isothiocyanate derivatives were in the same range. The difference by a factor around two of the apparent K_m_ value indicates that variations in the aromatic groups (benzyl vs phenetyl) do not affect much substrate recognition. Comparing all substrates, the highest K_*m*_ value was for CDNB but this is compensated by a better turnover number which is around 6–20 fold better than for the other substrates tested. Using PNP-butyrate as the second substrate, an apparent affinity of GSTF1 for GSH was determined. The K_m_ value is 97.6 ± 6.0 μM.

**Table 2:** **Kinetic parameters of PttGSTF1**.

	**BITC**	**PITC**	**CDNB**	**PNP-butyrate**	**HNE**	**CuOOH**	**HED**
**K_m_ (μM)**							
PttGSTF1	380.6 ± 43.7	148.9 ± 5.9	3065.6 ± 286.5	360.3 ± 32.2	67.1 ± 6.6	592.1 ± 51.6	ND
PttGSTF1 S13A	ND	ND	3313.9 ± 344.9	1510.9 ± 119.9	ND	ND	ND
PttGSTF1 S13C	ND	ND	ND	ND	ND	ND	33.7 ± 3.5
**k_cat_ (s^−1^)**							
PttGSTF1	0.70 ± 0.03	0.21 ± 0.02	4.20 ± 0.18	0.23 ± 0.40	0.21 ± 0.01	1.92 ± 0.04	ND
PttGSTF1 S13A	ND	ND	0.11 ± 0.01	0.060 ± 0.002	ND	ND	ND
PttGSTF1 S13C	ND	ND	ND	ND	ND	ND	0.040 ± 0.001
**k_cat_/K_m_ (M^−1^s^−1^)**							
PttGSTF1	1839.2 ± 87.2	1410.3 ± 19.0	1370.0 ± 3.6	661.9 ± 17.3	3141.6 ± 149.9	3245.7 ± 76.9	ND
PttGSTF1 S13A	ND	ND	35.4 ± 1.8	37.5 ± 1.0	ND	ND	ND
PttGSTF1 S13C	ND	ND	ND	ND	ND	ND	1124.7 ± 24.5

The apparent K_m_ values for all compounds were determined by varying substrate concentrations at a fixed saturating GSH concentration. The apparent K_m_ and k_cat_ values were calculated by non-linear regression using the Michaelis–Menten equation. Results are means ± S.D. (n = 3). ND means not detected. BITC, benzyl isothiocyanate; PITC, phenetyl isothiocyanate; CDNB, 1-chloro-2,4-dinitrobenzene; PNP-butyrate, 4-nitrophenyl butyrate; HNE, 4-hydroxy-2-nonenal; CuOOH, cumene hydroperoxide; HED, hydroxyethyl disulfide.

Contrary to Tau GSTs, GSTFs often proved to have peroxidase activities. For this reason, we have also tested cumene hydroperoxide (CuOOH) and tert-butyl hydroperoxide (t-BOOH). Whereas no activity was detected with t-BOOH, the catalytic efficiency obtained in steady-state conditions for the reduction of CuOOH into the corresponding alcohol is 3.2 × 10^3^ M^−1^s^−1^. This is in fact quite close to the value obtained for example with a mitochondrial Prx IIF from poplar, the role of which is assumed to significantly contribute to peroxide detoxification or signaling (Gama et al., 2007).

With most substrates, the substitution of the catalytic serine into alanine (PttGSTF1 S13A variant) generally led to a completely inactive enzyme. However, a residual activity was still observed with CDNB and PNP-butyrate, the catalytic efficiency being decreased by a factor of 40 and 20 respectively compared to the results obtained with PttGSTF1. Whereas this suggested that one or several residues other than the serine contribute to the decrease of the pKa of the thiol group of GSH, the PttGSTF1 S13C variant had no or negligible activity toward all these substrates. According to the mass spectrometry results, the reason may be the formation of a covalent adduct. Hence, this prompted us to investigative whether PttGSTF1 S13C has acquired properties similar to GSTs naturally having a cysteine residue in their active site signature by testing the thioltransferase activity using DHA and HED, two substrates usually employed for characterizing Grxs and cysteine-containing GSTs. As expected, PttGSTF1 had no activity both with HED and DHA. Concerning PttGSTF1 S13C, whereas no activity has been detected with DHA, a reasonably good catalytic efficiency (1.1 × 10^3^ M^−1^ s^−1^) was obtained with HED, essentially because of a good apparent affinity (K_m_ value of 33.7 μM).

Besides these classical assays, we sought to examine more unusual substrates/ligands that have been isolated with orthologous GSTF members i.e., auxin/indole-3-acetic acid (IAA) or a synthetic analog, 2,4-dichlorophenoxyacetic (2,4-D) (Bilang et al., 1993; Bilang and Sturm, 1995) and norharmane, indole-3-aldehyde and quercetin (Smith et al., 2003; Dixon et al., 2011). Hence, we investigated whether these compounds could constitute poplar GSTF1 substrates first by simply analyzing changes in the UV-visible spectra of each compounds as a function of time upon successive addition of GSH and PttGSTF1. However, we did not detect any significant spectral shifts (data not shown). Thinking that the glutathionylation may eventually not modify the absorption spectra of these molecules, the product of a reaction of several hours was analyzed by reverse phase-HPLC on a C18 column. However, no glutathionylated species can be separated and identified using this approach. Considering that some of these molecules may represent ligands and that the ligandin and catalytic sites in GSTs are generally overlapping at least partially, we have examined whether the addition of these molecules modulated PttGSTF1 activity. Despite using concentrations in the millimolar range, no effect was observed both using CDNB and PNP butyrate assays. We concluded that these compounds do not bind to PttGSTF1.

### The structures of PttGSTF1 and PttGSTF1 S13C in complex with GSH and MES reveal the residues participating to substrate binding

**Figure 5:**
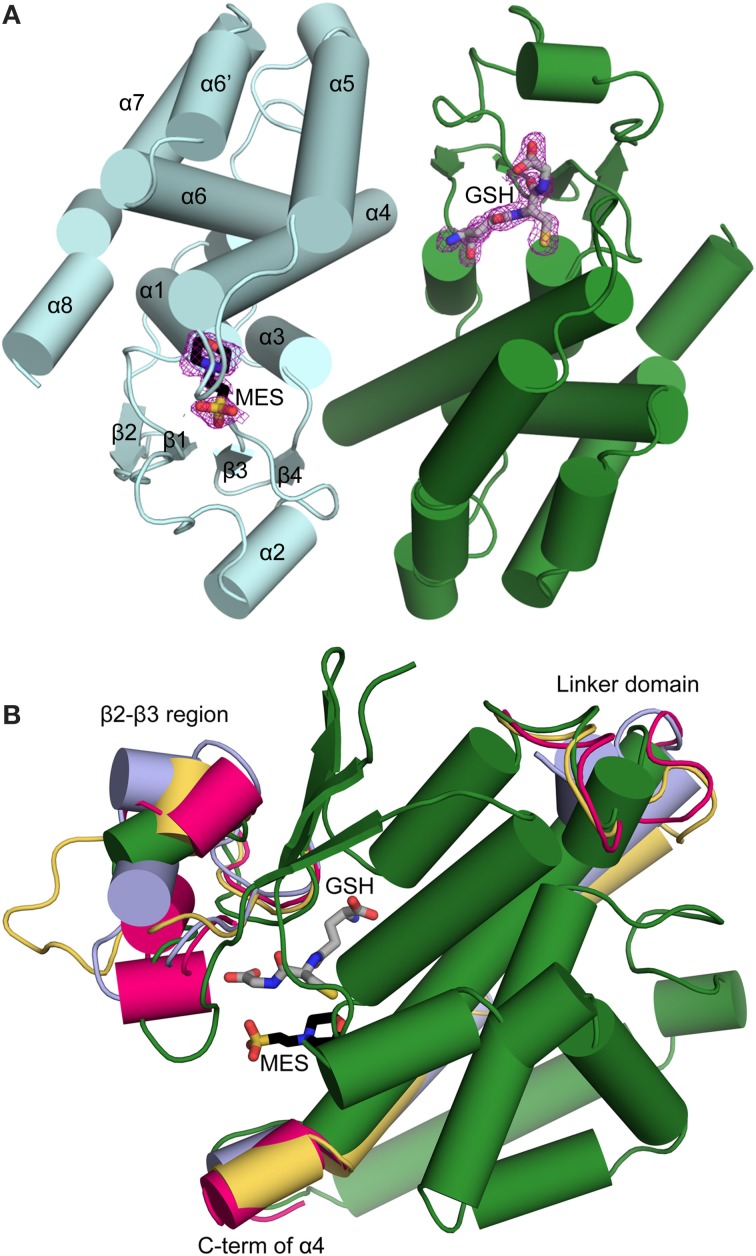
**Overall structure of PttGSTF1 and comparison with other plant GSTFs. (A)** Architecture of PttGSTF1 dimer. The MES molecule (black) is located in the H site, as shown in monomer A whereas the GSH molecule (gray) bound to the G site is depicted in monomer B. Monomers A and B are colored in light cyan and dark green respectively. Secondary structures are labeled only for monomer A for clarity. The GSH and MES molecules are shown as sticks and colored according to atom type (nitrogen, blue; oxygen, red; sulfur, yellow; and carbon, gray/black). Omit map (colored in purple) of contour level of 0.8 σ is shown around each bound ligand. It was built using the Composite omit map command of the Phenix software suite. **(B)** Superimposition of monomers of PttGSTF1 (dark green), *A. thaliana* GSTF2 (pink) and *Z. mays* GST-I (blue) and GST-III (yellow). Only noticeable secondary structural differences between GSTFs are shown for clarity. The GSH (gray) and MES (black) molecules highlight the putative positions of the G and H sites respectively. GSH and MES molecules are colored according to atom type and shown as sticks.

The crystallographic structures of PttGSTF1 and PttGSTF1 S13C, bound with ligands, have been obtained and refined to 1.5 and 1.8 Å resolutions (Table 1). The crystals belonged to the space group *P*2_1_2_1_2_1_, and the asymmetric unit consisted of one biological dimer (residues Ala2-Ala215 in both monomers, Root Mean Square Deviation of 0.18 Å for 175 superimposed Cα atoms). The analysis of the Fourier difference maps of PttGSTF1 revealed the presence of two ligands in the active site in each monomer: a glutathione molecule originating from the pre-treatment performed with an excess of GSH and a MES molecule present in the crystallization buffer. They are located respectively in the G and H sites (Figure 5A). Unless covalently bound, both ligands cannot occupy the active site simultaneously. Currently, we do not have any evidence for a GSH-conjugation reaction with MES nor data for any non-catalytic binding. Both glutathione and MES molecules were refined with complementary occupancies. In monomer A, the refined occupancies of glutathione and MES molecules were 58 and 42%, respectively. In monomer B, the corresponding refined occupancies were 71 and 29%, respectively. Therefore, PttGSTF1 structure can be described as two structures: PttGSTF1 in complex with glutathione and PttGSTF1 in complex with a MES molecule. Concerning PttGSTF1 S13C, the structure refinement confirmed that Cys13 is glutathionylated but this modification did not induce significant conformational changes in comparison to PttGSTF1 (RMSD of 0.33Å based on alignments of 350 Cα positions).

In order to understand possible differences among GSTF isoforms, a detailed comparison was performed with the three other GSTFs (AtGSTF2, maize GST-I and GST-III) whose structures are known (Reinemer et al., 1996; Neuefeind et al., 1997a,b; Prade et al., 1998). The AtGSTF2 structure was solved in complex with S-hexylglutathione or with an acetamide herbicide like molecule-glutathione conjugate, ZmGST-I was in complex with lactoylglutathione or an atrazine-glutathione conjugate, and ZmGST-III was in an apoform. Interestingly, PttGSTF1 belongs to a distinct, uncharacterized GSTF subgroup (Figure 2). A PttGSTF1 monomer consists of an N-terminal domain (β1α1β2α2β3β4α3) and a C-terminal domain composed of α-helices (α4α5α6α6′α7α8) (Figure 5A) as classically observed in most GST classes. As expected, structures of plant GSTFs superimposed relatively well with a mean RMSD of 0.92 Å. Prominent differences are nevertheless observed in three regions (Figure 5B). In PttGSTF1, an additional α-helix is observed in the segment between the strands β2 and β3 while others exhibit 1–3 short 3_10_-helices. This segment is involved in substrate binding and contains a conserved phenylalanine (Phe53 in PttGSTF1), which is assumed to be essential for dimerization (Prade et al., 1998). This phenylalanine represents the major inter-monomer contact, its side chain being buried in a hydrophobic pocket composed of Trp102, Thr105, Thr109, Val143, Ile146, and Tyr147 in PttGSTF1 and located between α4 and α5 of the other subunit. Interestingly, among the residues involved in the dimer interface, the hydrophobic ones are those that are the most conserved in PtGSTFs (Figure 1). Another variation concerns the length and conformation of the linker found between α3 and α4 helices and that connects the N- and C-terminal domains. Considering the variable length of the linker, such conformational differences were expected. However, the central residue of this connecting region, Leu88 in PttGSTF1, is highly conserved and adopts a superimposable position in all plant GSTF structures. Its side chain, wedging between helices α3 and α6, connects the two domains. The last noticeable difference is likely to be a class-specific feature of PttGSTF1 in which the absence of 3 residues found in other poplar GSTFs (Figure 1) shortens the α4 helix.

**Figure 6:**
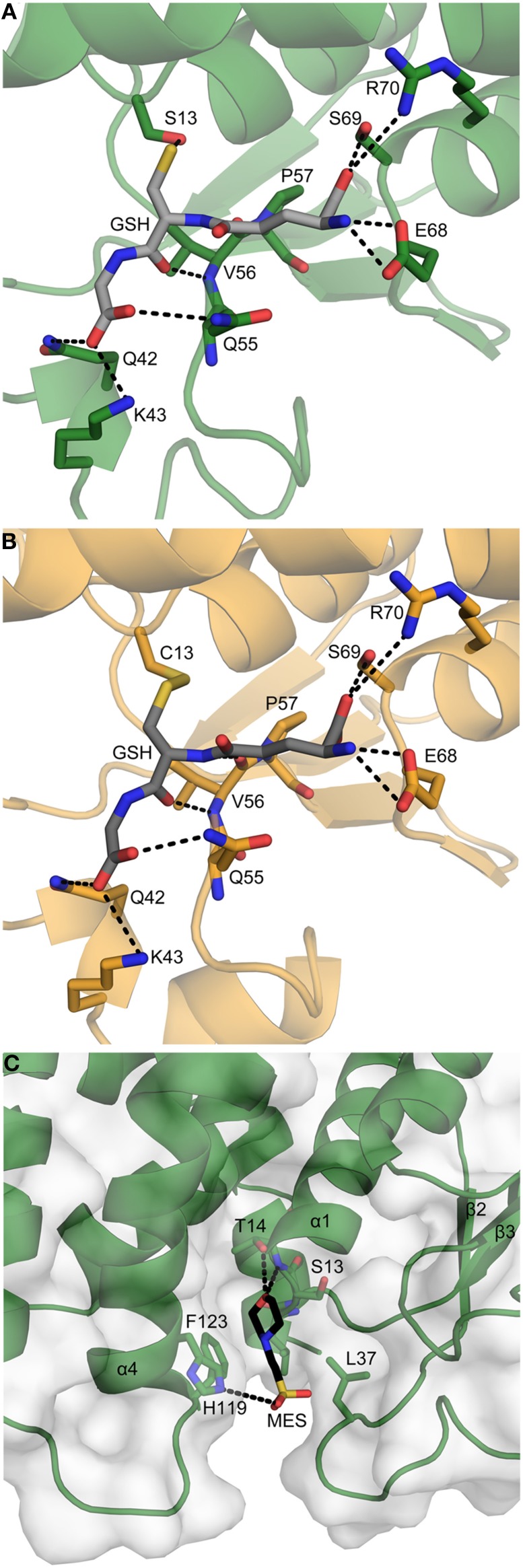
**Close up view of the G- and H-sites. (A,B)** Glutathione binding site of PttGSTF1 and PttGSTF1 S13C. **(C)** Electrophilic substrate binding site of PttGSTF1. The PttGSTF1 (dark green) and PttGSTF1 S13C (orange) monomers are shown in cartoon with a transparent molecular surface in **(C)**. Residues involved in the binding of GSH (gray) and MES (black) molecules are shown as sticks, labeled and colored according to atom type. Putative hydrophilic interactions between the substrates and the enzyme are shown as black dashed lines.

In PttGSTF1, a glutathione molecule is positioned in the G site groove which is mainly populated by polar residues from the N-terminal domain (Figure 6A). The Glu68, Ser69, and Arg70 residues, situated in the β4-α3 loop and in α3, stabilize the glutamyl group of GSH through hydrogen bonds and Coulomb interactions. The NH and carbonyl groups of the cysteinyl moiety are hydrogen-bonded to the backbone amino group of Val56 that precedes the invariant cis-Pro57 found in all GSTs and in all Trx superfamily members. The carboxylate of the glycinyl residue interacts with the side chains of Gln42, Lys43 and Gln55 found in the loops connecting β2-α2 and α2-β3. The thiol group of the cysteine of the GSH moiety is quasi-equidistant to the hydroxyl groups of Ser13 and Thr14 (3.2 and 3.4 Å respectively). According to mass spectrometry data, in the PttGSTF1 S13C variant, GSH is covalently bound to the modified residue (Cys13). Apart this difference, the same GSH-protein interactions are observed in both crystal structures (Figure 6B). With regard to the electrophilic substrate site, a MES molecule occupies the position adopted by other substrates in known GSTF structures. Hence, the H site is delimitated by residues from three regions: residues 12–14 found at the end of the β1-α1 loop and in α1, residues 36–40 that are part of the β2-α2 loop and residues 119–123 which are located in the C-terminal end of α4 (Figure 6C). The MES molecule is surrounded by the hydrophobic residues Leu12, Leu37 and Phe123. Moreover, the oxygen atom of the morpholino ring is hydrogen-bonded to the NH and OH groups of Thr14 and the sulfonic group forms a salt bridge with His119. However, the latter two residues are less conserved as compared to the three others suggesting that they might confer substrate specificities to PttGSTF1.

## Discussion

The existence of multigenic families is frequently explained by the functional divergence i.e., the acquirement of new or specific functions, appearing following gene duplication. With the complete sequencing of several plant genomes, it appeared that many species-specific duplication events occurred, leading to the expansion of the *GSTF* gene family. The maintenance of so many GSTF genes in the genomes (eight in poplar but up to *ca* 27 in some terrestrial plants) might be attributed for example (i) to a specific cellular/tissular expression associated to certain developmental stages or stress conditions, (ii) to specific subcellular localizations or (iii) to specific biochemical and structural characteristics. An additional layer of complexity and possible redundancy is the existence of several tens of GSTUs in plants which have quite similar enzymatic and biochemical properties. Indeed, owing to the presence of the same conserved serine residue, GSTUs also possess glutathionylation activities toward herbicides, safeners and several other cyclic/aromatic compounds. An intriguing example illustrating the possible redundancy between GSTUs and GSTFs is the fact that petunia *AN9*, a GSTF gene, and maize *Bz2*, a GSTU gene, can complement mutants for the other gene (Alfenito et al., 1998). Some differences can however be sometimes noticed. For instance, contrary to most GSTFs, GSTUs usually do not have peroxidase activity. However, redundancy could exist with other GST classes, notably the Theta GSTs that do have such a peroxidase activity.

In this study, we provide the first elements exploring the question of the redundancy among GSTF members and functions in poplar. Focusing on the particularities among poplar GSTFs that could explain the presence of eight genes, their putative subcellular localizations were first examined from the bioinformatic analysis of primary sequences. According to the absence of clear N- or C -terminal targeting sequences, all poplar GSTFs are predicted to be cytosolic proteins. This is generally in accordance with data obtained in other organisms either from translational GFP fusion as for several GSTFs of *Physcomitrella patens* (Liu et al., 2013) or from the absence of GSTF detection in studies of organellar proteomes. A plasma membrane localization was suggested for AtGSTF2 (Murphy et al., 2002) and a dual targeting in the cytosol and chloroplast was demonstrated for AtGSTF8 owing to the presence of an alternative transcription start site (Thatcher et al., 2007). However, only a few AtGSTF8 orthologs in other plant species have a similar extension. With regard to expression profiles, all poplar *GSTF* genes are redundantly expressed in some organs as leaves or reproductive organs. Moreover, the transcript levels are not necessarily correlated with protein levels. For instance, we did not detect GSTF1 transcripts in roots whereas quite important protein amounts were detected by western blot. It certainly illustrates the variations inherent to the plant developmental stages or to the fluctuations of environmental constraints as we have harvested our samples from a naturally growing tree and at different periods. Considering that many GSTFs could have similar cellular and subcellular expression territories, the difference should come from specific biochemical and/or structural properties. This parameter has been examined by producing PttGSTF1 as a recombinant protein and assessing its activity toward model substrates representing various types of biochemical activities as well as by solving the 3D structure of the first GSTF representative from a tree. Indeed, structures for only three GSTFs have been solved in the late 90's and nothing since that time.

As other GSTF members bearing a conserved serine in the active site motif, enzymatic analysis showed that GSTF1 possesses glutathione-conjugating activity toward structurally diverse substrates and glutathione peroxidase activity. The kinetic parameters of GSTF1 activity toward the model substrate CDNB (k_cat_/K_m_ of 1.3 × 10^3^ M^−1^s^−1^) are within the range of reported values for some GSTFs as *P. patens* GSTF1 (k_cat_/K_m_ of 1.5 × 10^3^ M^−1^s^−1^) (Liu et al., 2013) although important variations can be sometimes detected. *Triticum aestivum* GSTF1 exhibits a 50 fold higher catalytic efficiency (k_cat_/K_m_ of 7.2 × 10^4^ M^−1^s^−1^) (Cummins et al., 2003). While CDNB is an artificial substrate that may somehow mimic the structure of some herbicides and that is usually modified by all GSTFs, the other substrates used may be more physiologically relevant. BITC and PITC are representatives of a family of natural compounds found in Brassicaceae and produced by the enzymatic degradation of glucosinolates. Surprisingly, whereas glucosinolates are found in Arabidopsis, only a few Arabidopsis GSTF members among the 13 isoforms are able to catalyze conjugation reactions on BITC (Wagner et al., 2002; Nutricati et al., 2006; Dixon et al., 2009). The quite important turnover number obtained for the GSH-conjugation reaction of BITC by PttGSTF1 (k_cat_ of 0.70 s^−1^) indicates that poplar GSTF1 may have the particular ability to recognize related molecules. As a matter of comparison, higher turnover numbers, around 25 s^−1^, have been reported for *Homo sapiens* GST M1-1 or P1-1 both using BITC and PITC (Kolm et al., 1995). CuOOH is used as a molecule representative of bulky peroxides such as peroxidized lipids whereas HNE is a toxic aldehyde formed as a major end product of lipid peroxidation (Esterbauer et al., 1991). Both types of molecules have a dual function, being deleterious by promoting DNA damages or membrane protein inactivation, but at the same time, they represent signaling molecules. Whereas peroxide activity is systematically tested for GSTFs, the GSH-conjugation of HNE has been rarely evaluated. One example is the demonstration that a *Sorghum bicolor* B1/B2 GSTF heterodimer purified from shoots of fluxofenim-treated plants exhibits a catalytic efficiency about 15 fold higher (calculated k_cat_/K_m_ for this protein is around 2 × 10^4^ M^−1^s^−1^) than for PttGSTF1 (k_cat_/K_m_ of 1.3 × 10^3^ M^−1^s^−1^) (Gronwald and Plaisance, 1998). With regard to peroxides, most GSTFs tested so far, whatever their origin, exhibit a glutathione peroxidase activity. Compared to other characterized GSTFs, poplar GSTF1 possesses quite elevated peroxidase activity toward cumene hydroperoxide with a turnover number of 1.92 s^−1^ (Dixon et al., 2009). It is for instance in the same range as those reported for *Lolium rigidum* and *Alopecurus myosuroides* GSTF1 which are considered as highly active peroxidases (k_cat_ of 2.64 and 1.3 s^−1^ respectively) (Cummins et al., 2013). From a physiological perspective, it is worth noting that pathogen attacks are often accompanied by an oxidative stress that triggers, among other symptoms, lipid peroxidation. Also, important amounts of HNE are accumulated in *Phaseolus vulgaris* upon fungal infection by *Botrytis cinerea* (Muckenschnabel et al., 2002). With the known induction of *GSTF* genes by defense hormones or biotic stresses (Wagner et al., 2002), their known involvement in the synthesis of defense compounds as camalexin (Su et al., 2011), the peroxidase and GSH-conjugating HNE activities, it is conceivable that GSTF1 is involved in oxidative stress tolerance and/or oxidative signaling occurring in particular during pathogen or insect attacks. In fact, whereas *GSTF1* expression is induced in poplar attacked by the tent caterpillar *Malacosoma disstria* (Ralph et al., 2006) contrasting results have been obtained for GSTF1 in the case of rust infected poplars. Indeed *GSTF1* gene was found to be up-regulated at six dpi in a former study investigating gene expression in *Populus trichocarpa* × *P. deltoides* leaves infected by *Melampsora medusae* which represent a compatible interaction (Miranda et al., 2007). On the other hand, no regulation was detected when *Populus nigra* × *P. maximowiczii* leaves are infected by *M. medusae* or *M. larici-populina* (Azaiez et al., 2009) or when *P. trichocarpa* × *P. deltoides* leaves are infected by *M. larici-populina* either by a virulent (compatible) or an avirulent (incompatible) isolate (Rinaldi et al., 2007). According to the transcript measurements, no variation of GSTF1 protein level has been detected in *P. trichocarpa* × *P. deltoides* leaves during both compatible and incompatible reactions with *M. larici populina*. Here, the observed differences might simply be explained by differences in the poplar cultivars, rust isolates and time-points used in these independent studies, which altogether generate some specificity in these interactions. It would be informative to systematically analyze transcript and protein variations for all poplar GSTFs in different biotic interactions as done previously for example for the Arabidopsis or wheat GSTF families (Wagner et al., 2002; Cummins et al., 2003). To summarize this part, the biochemical and expression analyses demonstrated that, through its peroxidase and its GSH-conjugating activities, GSTF1 may have multiple roles notably related to xenobiotic detoxification or to oxidative stress tolerance both under biotic and abiotic constraints.

Contrary to other GSTFs, we have not observed an interaction or an activity with auxin and other heterocyclic compounds such as norharmane, indole-3-aldehyde and quercetin that were previously found to interact with other GSTFs and AtGSTF2 in particular (Bilang and Sturm, 1995; Smith et al., 2003; Dixon et al., 2011). This may indicate that PttGSTF1 has no ligandin function. In fact, several GSTFs for which ligandin function has been demonstrated, such as AN8 or Bz2, do not have the catalytic serine but an alanine instead in a AAxP motif. It does not mean however, that these GSTFs do not have catalytic functions. In fact, when the catalytic serine of PttGSTF1 was replaced by an alanine, the glutathionylation activity is not totally abolished as we would expect and a weak activity toward certain substrates was still measurable. This suggests that the catalytic serine is important but not mandatory for GSH-conjugating reactions and that residues other than the catalytic serine could be involved in glutathione activation. In support of this view, it has been reported that human GSTO1-1, a Cys-GST, loses deglutathionylation activity and acquires glutathionylation activity when the catalytic cysteine is replaced by an alanine (Whitbread et al., 2005). While Ser13 likely corresponds to the catalytic residue found in most GSTFs and is the primary candidate for GSH activation, the hydroxyl group of the adjacent Thr14 is found approximately at the same distance in the PttGSTF1 structure. Hence, it is tempting to conclude that it might substitute to Ser13, at least in its absence. It is worth noting that with the exception of some GSTF clades, the members of which have a proline, the catalytic serine is often followed by another Ser or Thr in most members of other clades (Figure 2). Interestingly, neither the serine nor the threonine is conserved in the clade containing poplar GSTF8 which harbors aliphatic residues at these positions (AACP signature). In this specific case, we could speculate that the cysteine found after the threonine position acts as the catalytic residue. Although this will have to be confirmed experimentally, it is interesting to note that poplar GSTF3 and F7 and their close orthologs also have a cysteine at this position, and that fungal Ure2p-like enzymes have an asparagine that was recently assumed to be important for catalysis (Thuillier et al., 2013). Overall, this suggests that all residues forming the active site signature and present around the N-terminal end of α1 could substitute to each other. Another proof of this assumption is that the PttGSTF1 S13C mutant lost its glutathione peroxidase and glutathionylating activity but acquired the capacity to perform deglutathionylation reaction toward HED, an activity typical of Cys-GSTs. Although the detected activity is weaker than the one obtained with naturally-existing Cys-GSTs (Lallement et al., 2014a), it shows that changing the nature of the catalytic residue is sufficient to determine the type of GST activity. Accordingly, when the catalytic cysteine of poplar Lambda GSTs is mutated into a serine, a shift from the original deglutathionylation to glutathionylation activity was observed (Lallement et al., 2014b).

Complementary to the biochemical and enzymatic analyses, the structural analysis should help understanding why GSTFs accept such diverse substrates but at the same time what are the fine differences that would generate substrate specificity. A comparison of poplar PttGSTF1 with AtGSTF2 structure does not point to dramatic structural changes. In fact, the glutathione binding site is in general not very different within a GST class but also among diverse GST classes. This is what we observed by superimposing GSTF structures. Rather, structural differences if any should come from variations in the H-site. However, owing to the lack of structures of GST in complex with their ligands, this H-site is often not very well defined. In the PttGSTF1 structure, the MES molecule, which likely mimics an H-site substrate (although it does not seem to be catalytically glutathionylated), is stabilized by five residues, Leu12, Thr14, Leu37, and His119 and Phe123. The Thr14 which is present in all poplar GSTFs except GSTF8, is in fact not found in other proteins whose structures are known, AtGSTF2 (SIAT signature), ZmGST-I (SWNL signature), and ZmGST-III (SPNV signature). Similarly, the His119 position is variable and it is occupied by an aromatic residue (Phe or Trp) in other poplar GSTFs as well as in AtGSTF2, ZmGST-I and ZmGST-III. Hence it is possible that these residues contribute to the recognition of specific substrates by PttGSTF1. In particular, the presence of His119 may be responsible for the binding of the MES molecule. On the contrary, the residues found at positions equivalent to Leu12, Leu37, and Phe123 in PttGSTF1 are also hydrophobic in most plant GSTFs and they are involved in the stabilization of the substrate in known structures of plant GSTFs in complex with herbicides. Thus, they seem to be critical for the electrophilic substrate recognition and they could constitute the core residues required for the general recognition of substrates. Supporting this view, it was shown that the Phe123 to Ile substitution in AtGSTF2 altered its ligand affinity and specificity (Dixon et al., 2011). Leu37 is found between β2 and β3, a region which is not well superimposable from one structure to another. For instance, five residues from this region are not visible in the electron density of the crystal structure of apo ZmGSTIII (Neuefeind et al., 1997b). A Phe35 modification in ZmGST-I (the residue equivalent to Leu37 in PttGSTF1) affects the enzyme affinity for its ligand (Axarli et al., 2004). Overall, this indicates that the β2-β3 region could be the protein area used by GSTFs to accommodate such a large spectrum of ligands/substrates. In GSTUs, the end of α4 helix and the C-terminal part are other regions that contribute to the correct positioning of the substrate in the H-site (Axarli et al., 2009). Similarly, the residues found at the end of α4 helix are also used by Lambda and Omega GSTs for substrate recognition which also involves the α4-α5 loop and a C-terminal helix (α9) which is specific to these two classes (Lallement et al., 2014b). In contrast, in GHR/Xi GSTs, proteins specialized in the reduction of glutathionylated quinones, no ample conformational change occurs upon substrate binding (Lallement et al., 2014c).

To conclude on these biochemical and structural analyses, it is conceivable that most GSTFs display a common set of enzymatic activities on typical substrates that is linked to the conservation of core residues. The persistence of closely related genes in single species may be explained by subtle sequence changes that confer the ability to the enzymes to accommodate specific substrates and thus to acquire specific functions. Hence, to address this question of the enzyme divergence and substrate specificity, isolating and identifying physiological GSTF substrates should become a priority as well as accumulating more 3D structures of GSTFs from poplar and other plants, alone or more importantly in complex with their physiological substrates.

## Author contributions

Henri Pégeot, Cha San Koh, Benjamin Petre, and Sandrine Mathiot performed the experiments under the supervision of Sébastien Duplessis, Arnaud Hecker, Claude Didierjean, and Nicolas Rouhier. All authors contributed to the writing of the manuscript, have read and approved the final manuscript.

### Conflict of interest statement

The authors declare that the research was conducted in the absence of any commercial or financial relationships that could be construed as a potential conflict of interest.
