# Supplementary material for: The poplar Phi class glutathione transferase: expression, activity and structure of GSTF1
Source: Front Plant Sci. 2014 Dec 23;5:712. doi: 10.3389/fpls.2014.00712 (PMC4274894; doi:10.3389/fpls.2014.00712)
Supplement: Supplementary file 1 [file DataSheet1.ZIP › Supplementary_Tables_2and 3.DOCX]

**Supplementary Material of the paper**

**The poplar phi class glutathione transferase: expression, activity and structure of GSTF1**

Henri Pégeot^1,2^, Chasan Koh^3,4^, Benjamin Petre^1,2,¥^, Sandrine Mathiot^3,4^, Sébastien Duplessis^1,2^, Arnaud Hecker^1,2^, Claude Didierjean^3,4^, Nicolas Rouhier^1,2*^

**Supplementary Table 2.** Primers used in this study for RT-PCR, cloning and site-directed mutagenesis experiments

| **Name** | **Sequence** |
| --- | --- |
| RT-PCR experiments |  |
| *Pt-GSTF1* (*Potri. 002G015100*) |  |
| Pt-GSTF1-RT-For | 5’-CCCCCCATGGCTACTCCGGTGACTATT-3’ |
| Pt-GSTF1-RT-Rev | 5’-ATTTGCTTTTGATAAGATG-3’ |
| *Pt-GSTF2* (*Potri.002G015200*) |  |
| Pt-GSTF2-RT-For | 5'-CCCCCCATGGCAACTCCAGTGAAGGTG-3' |
| Pt-GSTF2-RT-Rev | 5-'TGATGCCCTTTCTAATGGG-3' |
| *Pt-GSTF3* (*Potri.014G132200*) |  |
| PtGSTF3-RT-For | 5'-CTCTCGTGTTCTGACTTGTCTG-3' |
| PtGSTF3-RT-Rev | 5'-TGCTATCGCAGGGTGGAATTGT-3' |
| *Pt-GSTF4* (*Potri.T035400*) |  |
| PtGSTF4-RT-For | 5'-TAAAGTCCACGGAAGCACCC-3' |
| PtGSTF4-RT-Rev | 5'-CTCCATCCACACTGATAA-3' |
| *Pt-GSTF5* (*Potri.T035300*) |  |
| PtGSTF5-RT-For | 5'-GAAACTCCATGGAAGCGTTT-3' |
| PtGSTF5/6-RT-Rev | 5'-TTCTTGCCATACCAAAAT-3' |
| *Pt-GSTF6* (*Potri.T035100*) |  |
| PtGSTF6-RT-For | 5'-GAAACTCCATGGAACCCCTA-3' |
| PtGSTF5/6-RT-Rev | 5'-TTCTTGCCATACCAAAAT-3' |
| *Pt-GSTF7* (*Potri.017G138800*) |  |
| PtGSTF7-RT-For | 5'-CTCTCGCGTGCTGACATGC-3' |
| PtGSTF7-RT-Rev | 5'-TGCTATCGCAGGGTTATATCGG-3' |
| *Pt-GSTF8* (*Potri.T035000*) |  |
| PtGSTF8-RT-For | 5'-TGCCCACAAAGGGTCATGGCT-3' |
| PtGSTF8-RT-Rev | 5'-GAACACCAGATCATTGAAGTTG-3' |
| Pt-UBQ (*Potri.015G013600*) |  |
| Pt-UBQ-RT-For | 3’-GCAGGGAAACAGTGAGGAAGG-3’ |
| Pt-UBQ-RT-Rev | 5’-GCTCTGGACTCACGAGGACAG-3’ |
|  |  |
| Cloning in pET-3d expression vector |  |
| Pt-GSTF1-*Nco*I-For | 5’-CCCCCCATGGCTACTCCGGTGACTATT-3’ |
| Pt-GSTF1-*Bam*HI-Rev | 5’-CCCCGGATCCTCAAGCATTTTTCCTCAT-3’ |
|  |  |
| Mutagenesis |  |
| Pt-GSTF1-S13C-For | 5’- TACGGGCCACCATTGTGCACGGCAGTGTCGAGA-3’ |
| Pt-GSTF1-S13C-Rev | 5’- TCTCGACACTGCCGTGCACAATGGTGGCCCGTA-3’ |
| Pt-GSTF1-S13A-For | 5’-TACGGGCCACCATTGGCCACGGCAGTGTCGAGA-3’ |
| Pt-GSTF1-S13A-Rev | 5’-TCTCGACACTGCCGTGGCCAATGGTGGCCCGTA-3’ |
|  |  |

**Supplementary Table 3.** Molecular mass analysis of native PtGSTF1, PtGSTF1 S13A and S13C by mass spectrometry and analytical gel filtration

| **Protein** | **Theoretical mass (Da)** | **Native (Da)** | **Mass determined by FPLC (Da)** |
| --- | --- | --- | --- |
| Pt-GSTF1 | 24320.7 | 24192 (- Met) | 47600 (Dimer) |
| Pt-GSTF1 S13C | 24336.8 | 24511 (- Met + GSH) | 45000 (Dimer) |
| Pt-GSTF1 S13A | 24304.7 | 24172 (- Met) | 47200 (Dimer) |
