# Supplementary material for: The poplar Phi class glutathione transferase: expression, activity and structure of GSTF1
Source: Front Plant Sci. 2014 Dec 23;5:712. doi: 10.3389/fpls.2014.00712 (PMC4274894; doi:10.3389/fpls.2014.00712)
Supplement: Supplementary file 1 [file DataSheet1.ZIP › Supplementary_Table_1.DOCX]

**Supplementary Material of the paper**

**The poplar phi class glutathione transferase: expression, activity and structure of GSTF1**

Henri Pégeot^1,2^, Chasan Koh^3,4^, Benjamin Petre^1,2,¥^, Sandrine Mathiot^3,4^, Sébastien Duplessis^1,2^, Arnaud Hecker^1,2^, Claude Didierjean^3,4^, Nicolas Rouhier^1,2*^

**Supplementary Table 1. Amino acid GSTF sequences in photosynthetic organisms**

GSTF sequences used to construct the phylogenetic tree shown in Figure 2 and retrieved from *Chlamydomonas reinhardtii, Chlorella sp. NC64A, Coccomyxa subellipsoidea C-169, Micromonas pusilla CCMP1545, Micromonas pusilla RCC299, Ostreococcus lucimarinus, Volvox carteri, Physcomitrella patens, Selaginella moellendorffii, Brachypodium distachyon, Oryza sativa, Panicum virgatum, Setaria italica, Sorghum bicolor, Zea mays, Aquilegia coerulea, Mimulus guttatus, Solanum lycopersicum, Solanum tuberosum, Vitis vinifera, Eucalyptus grandis, Populus trichocarpa, Carica papaya, Gossypium raimondii, Theobroma cacao, Arabidopsis lyrata, Arabidopsis thaliana, Boechera stricta, Brassica rapa FPsc, Capsella grandiflora, Capsella rubella, Eutrema salsugineum, Citrus sinensis, Citrus clementina, Linum usitatissimum, Manihot esculenta, Ricinus communis, Cucumis sativus, Fragaria vesca, Glycine max, Malus domestica, Medicago truncatula, Phaseolus vulgaris, Prunus persica.*

**The data is available in the file: Supplementary_Table_1.XLSX**
